# MY PARENT’S BODY IS SACRED: LATINO PERSPECTIVES ON BRAIN DONATION FOR ALZHEIMER’S DISEASE RESEARCH

**DOI:** 10.1093/geroni/igz038.1524

**Published:** 2019-11-08

**Authors:** Yadira Montoya, Guilherme M Balbim, Crystal M Glover, David X Marquez

**Affiliations:** 1 NORC at the University of Chicago, Chicago, Illinois, United States; 2 University of Illinois at Chicago, Chicago, Illinois, United States; 3 Rush Alzheimer’s Disease Center, Chicago, Illinois, United States

## Abstract

Brain donation is a critical part of advancing research addressing Alzheimer’s disease and related dementias (ADRD). Latinos are at a higher risk of developing ADRD compared to non-Latino Whites. However, there is limited knowledge regarding causes and mechanisms related to ADRD health disparities among Latinos partially due to lower research participation and brain donation rates. Family members play a pivotal role in increasing brain donation rates, particularly, among underrepresented groups. In this study, we examine the perceptions of brain donation among adult children of older Latinos. We invited Latino men and women (N=15) with a parental-figure who was 65 years and over to participate in one of three focus groups. During the focus groups, participants discussed the meaning of brain donation for research, reasons to donate or not, and their reactions to the possibility of their parental-figure being a brain donor. All focus groups were audio-recorded and transcribed with transcripts used for data analysis. We used a Grounded Theory Approach to analyze focus group data. Results yielded three themes: (1) social and cultural factors influencing a family’s willingness to support organ donation; (2) lack of knowledge about the brain donation process; and (3) recommendations for engaging more Latinos in ADRD research and brain donation. Findings provide insight into how family participation may facilitate increased brain donation rates in ADRD studies among older Latinos. A main recommendation for researchers is to adopt a family-centered approach throughout the research process with a focus on addressing information gaps - from recruitment to dissemination.

